# Cutaneous metastasis of renal cell carcinoma: Fine needle aspiration provides rapid diagnosis

**DOI:** 10.1002/ccr3.1943

**Published:** 2018-12-14

**Authors:** Rusella Mirza, Steven Ellsworth, Judy King, Guillermo Sangster, Mingxia Shi

**Affiliations:** ^1^ Department of Pathology and Translational Pathology Louisiana State University Health Science Center Shreveport Louisiana; ^2^ Department of Clinical Radiology and Anesthesiology Louisiana State University Health Science Center Shreveport Louisiana

**Keywords:** cutaneous, fine needle aspiration, metastasis, renal cell carcinoma

## Abstract

The diagnosis of cutaneous metastasis of renal cell carcinoma is challenging in a young person in absence of a prior history of cancer. In such situation, fine needle aspiration alone as a minimally invasive procedure can provide rapid, accurate and cost effective diagnosis, even in case of unknown primary.

## 1 INTRODUCTION

Renal cell carcinoma (RCC) accounts for about 3% of all adult malignancy and is more common in men. The classic presentation is flank pain, hematuria and palpable flank mass. More than 70% of RCC are detected as an incidental finding. RCC has strong tendency to metastasize to almost any organ and about 30% of the cases present with metastasis.^1^ According to a retrospective study with 306 cases of renal carcinoma, the cutaneous metastasis was found in only about 3.3% cases.^2^ In most of the cases the distant metastasis was detected after nephrectomy secondary to RCC. There are relatively few reports of cutaneous RCC as an initial presentation. Mandal and Porter have reported two separate cases of cutaneous RCC as an initial presentation without concurrent renal symptoms.^3^, ^4^ In both cases, the skin nodules were being treated for other common differentials such as abscess or lymphoma. Due to absence of obvious renal symptoms, metastatic RCC was low in differential. Skin biopsy provided definitive diagnosis to those patients. Here, we report a cutaneous RCC case without any significant prior medical history. We demonstrate the usefulness of the fine needle aspiration (FNA) for the rapid and accurate diagnosis of RCC with cutaneous metastasis.

## 2 CASE PRESENTATION

**Figure 1:**
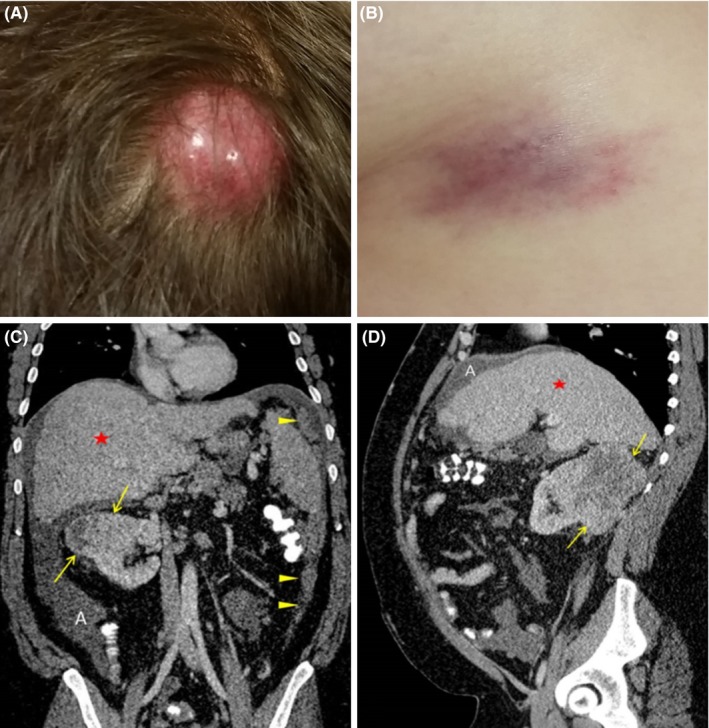
Skin lesions demonstrate, a nodular lesion at scalp (A) and a flat skin lesion at chest wall (B). Multiplanar coronal (C) and sagittal (D) contrast‐enhanced CT of the abdomen demonstrate a heterogeneous solid enhancing mass in the right upper renal pole (arrows) causing anatomic distortion at the renal parenchyma. Malignant peritoneal implants (arrowhead) and ascites (A) are noted. A cirrhotic liver is incidentally seen (*)

Our patient is a 41‐year‐old white male with no known past medical history of renal cell carcinoma presented with skin lesions on his scalp, chest and back for about one month. He was treated for cyst with Bactrim by his primary care physician without having any response. Upon examination, the lesions at scalp and back were found as round, raised, and firm mass measuring 2.0 × 2.0 × 1.5 cm. The chest lesion was flat (2.0 × 1.5 cm) with a palpable nodule underneath it (Figure 1A,B). All three lesions were violaceous and non‐tender. He also reported an intermittent sharp right‐sided abdominal pain for last one month. He denied any hematuria and weight loss. Lab works revealed normal CBC with increased creatinine (1.4 mg/dL). CT abdomen, chest and bone scan demonstrated a large heterogeneous exophytic mass of the upper right kidney measuring 11.0 × 11.0 × 10.0 cm (Figure 1C,D). He had mild ascites with multiple nodules in the posterior peritoneal wall, in lung and liver. Lymphadenopathy and lytic bone lesions were also noted. The cytopathology team was consulted for the rapid interpretation of FNA from the skin lesion of the chest wall. The patient was consented for the procedure and for the publication.

**Figure 2:**
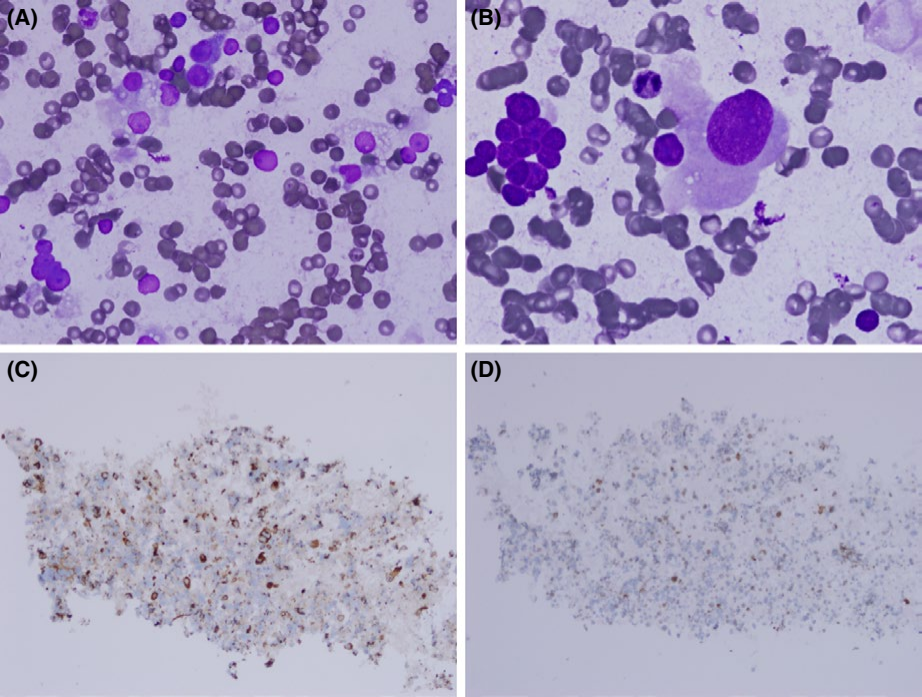
FNA smear of skin lesion showing mix population of cells with abundant wispy cytoplasm, round and naked nuclei with prominent nucleoli (A), Few large cells with less vacuolated cytoplasm (B), cell block is positive for AE1/AE3 (C) and for Pax8 (D). The magnification for A, B, C, and D is x40, x60, x10, and x10, respectively

Diff Quick preparation of FNA smear was hypercellular, with a mixture of discohesive and cluster of cells (Figure 2A,B). The tumor cells had low nuclear to cytoplasmic (N/C) ratio, eccentrically placed round nucleus with prominent nucleoli. Some cells were large in size with abundant finely granular and less vacuolated cytoplasm. Others were smaller with abundant vacuolated, wispy cytoplasm. About 60% of smear was composed of naked nuclei with prominent nucleoli. Our rapid interpretation was reported as “malignant cells present, favor renal cell carcinoma”. Tumor cells in the cell block were positive for Pax8 and AE1/AE3 (Figure 2C,D) by immunohistochemistry.

**Figure 3:**
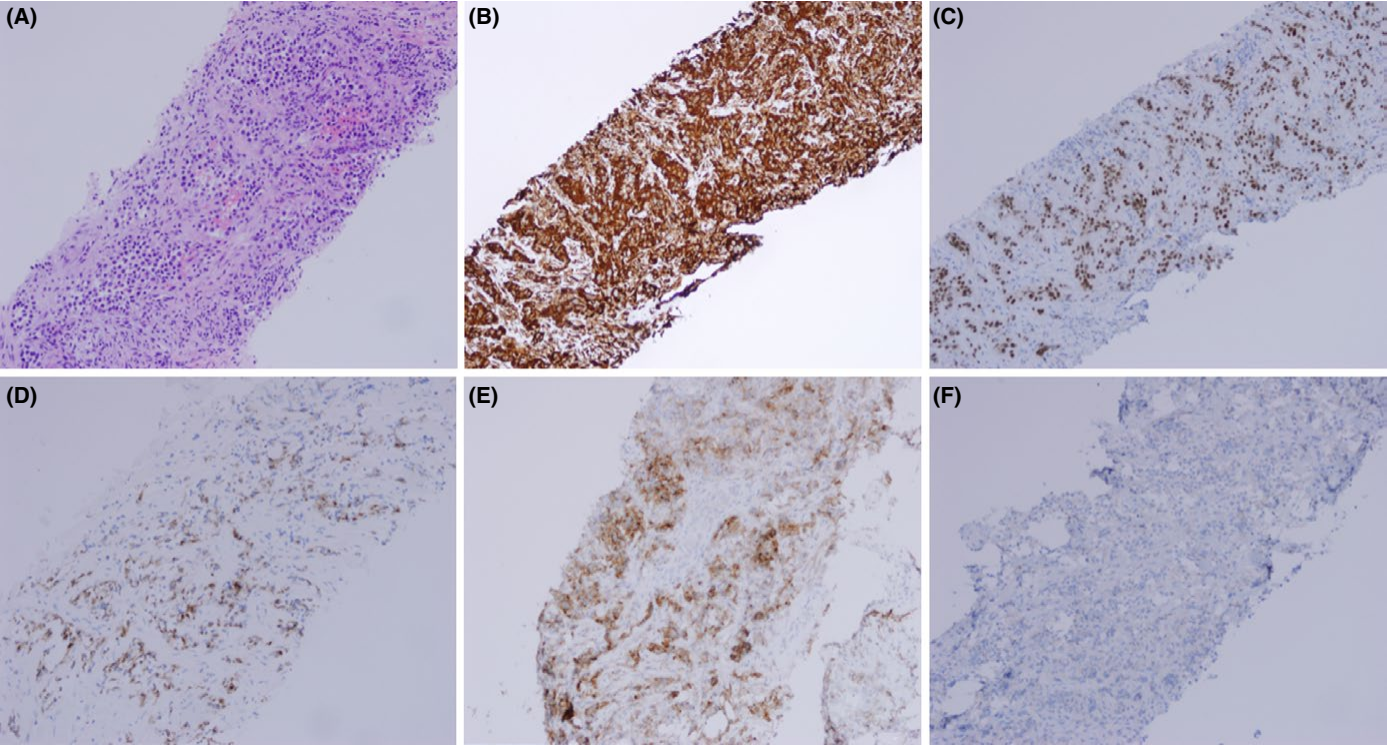
Core biopsy demonstrating infiltrating pattern of tumor cells with H&E stain (A), tumor cells are strongly positive for vimentin (B), Pax8 (C), RCC (D), CD10 (E) and negative for CK7 (F). The magnification for A, B, C is x10 and for D, E, and F is x20

Two core biopsies were collected concurrently with the FNA from the same skin lesion. Core biopsy demonstrated sheets of tumor cells infiltrating the underlying tissue. The tumor cells had similar cytomorphology to those observed in the FNA smear (Figure 3A). Tumor cells were positive for Pax8, RCC, vimentin, CD10 and negative for Ck7 (Figure 3). Based on the histomorphology and the immunohistochemistry findings, a diagnosis of metastatic clear cell renal cell carcinoma was made.

## 3 DISCUSSION

Cutaneous metastasis of RCC is a rare entity. Diagnosing cutaneous RCC in a young person without prior history is challenging. The helpful features in this case included the number of skin lesion, their violaceous color, the consistency, and the growth pattern. A rapidly growing mass which is not responding with conventional therapy should raise a concern and a biopsy or fine needle aspiration can be beneficial.^3^, ^4^ In most of the cases of cutaneous RCC are well circumscribed and non tender nodules.^3^, ^5^ A flat skin lesion can also be seen; however, a round mass may be noted underneath the skin, as we have observed in our patient.

Our FNA smear was hypercellular with tumor cells that preserved the cytomorphological characteristics specific for the renal cell carcinoma. Moreover, we have observed the characteristics of high grade tumor cells such as discohesiveness, less vacuolated cytoplasm and prominent nucleoli.^6^ Thus the cytomorphology was not only specific for the diagnosis but also was helpful for the grading of RCC. Differential diagnosis including fibrohistocytic lesion and lymphoma were considered due to appearance of histocytoid cells and discohesive naked nuclei. The neoplastic cells were strongly positive for Pax8, vimentin, RCC, CD10, AE1/AE3 and were negative for CD56 and CD45 which favored the diagnosis of clear cell carcinoma.

We also have received core biopsies which gave us the opportunity to compare the histology with the FNA smear. Core biopsy preserved the tissue architecture and provided more specimens for the further clarification of the lesion. However, FNA is less invasive than core biopsy. It was demonstrated by few other groups that cutaneous RCC was diagnosed by FNA.^5^, ^7^ Here we concur with others that in a proper setting FNA alone as a minimally invasive procedure can provide rapid, accurate and cost effective diagnosis of cutaneous metastasis of RCC even in case of unknown primary.

## CONFLICT OF INTEREST

None declared.

## AUTHOR CONTRIBUTION

RM and MS: Manuscript writing and figure preparation; SE and JK: Writing and editing; GS: Figure preparation.

## References

[1] Sountoulides P , Metaxa L , Cindolo L . Atypical presentations and rare metastatic sites of renal cell carcinoma: a review of case reports. J Med Case Rep. 2011;5:429. 21888643 10.1186/1752-1947-5-429 PMC3177931

[2] Dorairajan LN , Hemal AK , Aron M , et al. Cutaneous metastases in renal cell carcinoma. Urol Int. 1999;63(3):164‐167. 10738187 10.1159/000030440

[3] Mandal A , Littler Y , Libertiny G . Asymptomatic renal cell carcinoma with metastesis to the skin and duodenum: a case report and review of the literature. Bio Med J Case Rep. 2012 10.1136/bcr.02.2012.5764 PMC4542881 22684828

[4] Porter NA , Anderson HL , Al‐Dujaily S . Renal cell carcinoma presenting as a solitary cutaneous facial metastasis: case report and review of the literature. BioMed Central. 2006;3:27. 10.1186/1477-7800-3-27 PMC1578574 16968548

[5] Syriac S , Boergert S , Ganesan S . FNA diagnosis of cutaneous metastasis of renal cell carcinoma with initial presentation as scalp lesion. Annal Clin Cytotol Pathol. 2016;2(1):1015.

[6] Cibas ES , Ducatman BS . Cytology diagnostic principles and clinical correlates, 4th edn, Chapter 15: 423 Philadelphia, PA: Elsevier; 2013.

[7] Singh A , Mohan G , Chatuvedi S , Khan SA . Cytodignosis of a cutaneous clear cell malignancy: Metastatic renal cell carcinoma on chin. J Clin Diagn Res. 2016;10(1):ED12‐4. 10.7860/JCDR/2016/15869.7100 PMC4740607 26894079

